# Advanced subclinical atherosclerosis: A novel category within the cardiovascular risk continuum with distinct treatment implications

**DOI:** 10.1016/j.ajpc.2022.100456

**Published:** 2022-12-24

**Authors:** Michael J. Blaha, Magdy Abdelhamid, Francesca Santilli, Zhongwei Shi, Dirk Sibbing

**Affiliations:** a Johns Hopkins Ciccarone Center for the Prevention of Cardiovascular Disease, Blalock 524D1, 600 N. Wolfe Street, Baltimore, MD 21287, USA; b Department of Cardiovascular Medicine, Faculty of Medicine, Kasr Al Ainy, Cairo University, Egypt; c Department of Medicine and Aging and Center for Advanced Studies and Technology, University of Chieti, Chieti, Italy; d Ruijin Hospital, Shanghai Jiao Tong University School of Medicine, Shanghai, China; e Ludwig-Maximilians University (LMU), Germany and Privatklinik Lauterbacher Mühle am Ostersee, Munich, Seeshaupt, Germany

**Keywords:** Cardiovascular disease, Primary prevention, Subclinical atherosclerosis, Coronary artery calcium, Risk assessment, Education

## Abstract

Traditionally, guidelines divide patients into primary and secondary prevention for atherosclerotic cardiovascular disease (ASCVD) risk management. However, the modern understanding of the biological progression of atherosclerosis is inconsistent with this binary approach. Therefore, a new approach demonstrating both atherosclerosis and ASCVD risk as a continuum is needed to give clinicians a framework for better matching risk and intensity of therapy. Advances in coronary imaging have most clearly brought this problem into view, as for example coronary artery calcium (CAC) scoring has shown that some individuals in the primary prevention have equal or higher ASCVD risk as certain subgroups in secondary prevention. This article introduces “advanced subclinical atherosclerosis” as a new and distinct clinical group that sits between the traditional groups of primary and secondary prevention. Importantly, this article also introduces a new graphic to visualize this intermediate population that is explicitly based on plaque burden. The aim of the graphic is both to educate and to allow for better identification of a patient's cardiovascular risk and guide more effective risk-based management.

Cardiovascular diseases (CVD) remain the leading cause of disease burden in the world, with the age-standardized rate of CVD rising in some high-income countries [1]. Although the medical and surgical management of patients after an ischemic event has improved immensely, population-wide prevention of ischemic events lags behind. This is due in part to our limited ability to both identify patients at high risk and to deliver aggressive disease management. For example, we still largely rely on the assessment of traditional risk factors using population-based risk calculators such as the atherosclerotic cardiovascular disease (ASCVD) Risk Estimator [2] and SCORE 2 [3]. Outputs from risk calculators may overestimate or underestimate the risk associated with certain groups of patients, including those older patients without risk factors and young adults with a family history of CVD [4] or younger patients with metabolic syndrome en route to type 2 diabetes [5]. Furthermore, results from disease risk calculators are often difficult for end users to understand [6] and therefore to act upon.

There is growing evidence that an increased risk of CVD is present long before an acute ischemic event, and not infrequently before traditional risk factors are even detected [7]. As an example, impaired glucose tolerance is associated with a 20–30% increased risk of developing CVD [8] in the absence of overt type 2 diabetes. This increase in risk is apparent with higher glycated hemoglobin (HbA1c) in people within the so-called normoglycemic range [9]. This raises the questions: are current approaches to identifying high-risk patients flawed? What are we missing?

The pathophysiology of atherosclerotic disease is complex and progressive in nature. It develops silently throughout different vascular territories long before a stenosis reaches functional relevance or an ischemic event occurs [10], [11], [12]. Many studies have shown that the majority of coronary events happen in patients not previously considered high risk, who do not have known obstructive coronary artery disease, and/or whose functional tests remain normal [12], [13], [14]. A review conducted across 1,475 patients who experienced a myocardial infarction at age ≤50 years, found that more than 50% were considered low risk immediately before the event [12,14].

The use of noninvasive imaging techniques (coronary artery calcium [CAC] score and coronary computed tomography angiography [CCTA]) can refine the risk category that is defined by traditional algorithms alone [15]. Use of the CAC score is based on the understanding that calcifications of the coronary arteries are not a passive process but pathognomonic of evolving coronary atherosclerosis. Coronary calcification is nearly universal in all patients with documented coronary artery disease and its development is closely related to early aging (starting typically at >30–40 years of age [4]), vascular injury, inflammation and repair, and cardiovascular risk factors such as metabolic syndrome, dyslipidemia, tobacco use, hypertension, chronic kidney disease, high C-reactive protein levels, and high lipoprotein(a) [16]. Several large observational studies and reviews have shown that CAC score predicts future cardiovascular events and can be used to accurately classify patients into low-risk and high-risk categories [17,18]. Major guidelines recommend the use of CAC scoring and a recent paper provides guidance on determining the appropriate age to initiate clinical CAC testing [19].

However, as part of the total plaque volume comprises nondetectable noncalcified tissue, the CAC score may underestimate total coronary atherosclerotic plaque burden in select individuals, particularly younger people. Furthermore, the relationship of coronary calcification with significant stenosis is variable, as obstructive plaques can occur at sites with limited calcium and extensive calcific deposits can be observed without stenosis [15]. As imaging for early heart disease improves, new studies are painting an even clearer picture of subclinical atherosclerosis. For example, while a recent general population-based study of asymptomatic adults reported a tight association between total atherosclerosis detected by CCTA and increasing CAC score – all people with a CAC score >400 had atherosclerosis and 45.7% had significant stenosis on CCTA [20] – importantly, 5.5% of those with a CAC score of 0 had atherosclerosis and 0.4% had significant stenosis, and 10% of intermediate-risk patients with a CAC score of 0 had coronary atherosclerosis by CCTA. Equally remarkable is that 58% of the population had absolutely no plaque that could be detected by either CAC score or CCTA [20]. Another large community-based study of asymptomatic individuals noted that 16% of those with a CAC score of 0 had some plaque and over 2% had high-risk plaque features [21]. However, 51% of this primary prevention population had absolutely no plaque despite suboptimal risk factor levels. A systematic review and meta-analysis of 19 studies noted that 45% of patients who presented for work-up of acute chest pain had a CAC score of 0. In this review, the negative predictive values for a CAC score of 0 ruling out obstructive coronary artery disease (CAD) were 97% and 98% for stable and acute chest pain, respectively [22].

These studies documented marked heterogeneity of plaque burden have prompted many clinicians to challenge the reliance on traditional risk factors and the current clinical dichotomy of primary and secondary prevention. There is growing recognition of an intermediate population, a group of patients between those who are currently considered the primary prevention population and those who have suffered an ischemic event, the secondary prevention population. In this article, we posit that this intermediate population is best described as those with “advanced subclinical atherosclerosis.” This highly descriptive term is commonly understandable for patients and points to a new and distinct, yet highly prevalent, patient population. We believe that other terms such as “primary and a half prevention” [23] blend traditional concepts and fail to concretely describe a new population or concept. Naming this population is critically important; if clinical guidelines or clinical trials are to target such a population, it needs a descriptive name that can be clearly defined. A clear distinction between the primary prevention population and those with advanced subclinical atherosclerosis may also help to drive engagement in lifestyle changes that would prevent people from moving into this higher-risk population.

In order to effectively control the rise of CVD, there is an increasing need to identify this intermediate group of patients with advanced subclinical atherosclerosis and to manage their condition appropriately. This is even more important as our therapies for reducing CVD risk have improved. Our most intensive preventive treatments are rarely considered for patients with extensive but nonobstructive coronary artery disease [13,24]. Even though the most recent European Society of Cardiology/European Atherosclerosis Society guideline begins to incorporate this philosophy, the definition of high-risk populations and attendant recommendations are generally restricted to those in whom aggressive statin treatment is recommended [25]. Future recommendations should be based on the notion that event rates can be similar in high-risk primary and stable secondary prevention patients, and that our most effective treatments should not be reserved for secondary prevention alone [26].

Two important changes can be implemented to overcome the barriers to achieving better identification of patients with subclinical atherosclerosis and providing appropriate management. The first involves increasing the efficiency of patient identification, and the second involves motivating physicians and patients to engage in the most appropriate management of risk factors and the use of preventive medication outside of the traditional binary framework of primary/primordial and secondary prevention. Lifestyle changes are difficult for patients to maintain, Lp(a) is largely genetically determined, and target blood pressure, low-density lipoprotein (LDL) cholesterol levels, and HbA1c are not achieved in the majority of patients [27,28], despite the existence of numerous algorithms and treatment pathways that are designed to simplify the treatment choices. However, the impact of a high CAC score has been shown to positively impact the initiation and maintenance of preventive treatments and lifestyle changes for up to 10 years [17,29].

Graphics can play a vital role in communicating healthcare messages by linking cause and effect in complex conditions in a way that is easier to understand than text. Visual representations can increase attention to and recall of information when compared with text alone and may generate an emotional response that could then be related to health behaviors [30]. We reviewed the graphics developed so far describing the cardiovascular risk continuum and believe that there is a gap in the current educational literature [31], [32], [33], [34], [35]. Our international author group could not find a single graphic that fully encapsulates what we see as the atherosclerotic cardiovascular risk continuum across a person's lifespan.

Here, we introduce a new conceptual graphic to describe the many interlinked and progressive pathophysiological processes involved in atherosclerosis over its natural history (Fig. 1). This graphic illustrates the continuum of atherosclerosis (spanning primary and secondary prevention) and how patients at different points along this continuum may be at greater risk of an ischemic event than is apparent from traditional assessment of their risk factors. Although other authors have provided graphical representations of a disease continuum, these focus on stenosis and the ischemic event as the terminal event in the process. This new graphic is among the first to clearly show that cardiovascular risk progression is not strictly linear, in that some patients develop little or no atherosclerosis, while others can, and do, have sudden ischemic events even though they may be asymptomatic with nonobstructive disease. The graphic communicates how their risk post-event is directly influenced by the ongoing progression of underlying atherosclerosis in other vessels. It also attempts to explain why some patients who receive successful revascularization and aggressive treatment have a lower risk of a subsequent event than patients who have not yet had an event but have a high plaque burden (i.e. can be lower risk than the advanced subclinical atherosclerosis population), while some patients continue to progress to a “very high risk” status.

**Fig. 1:**
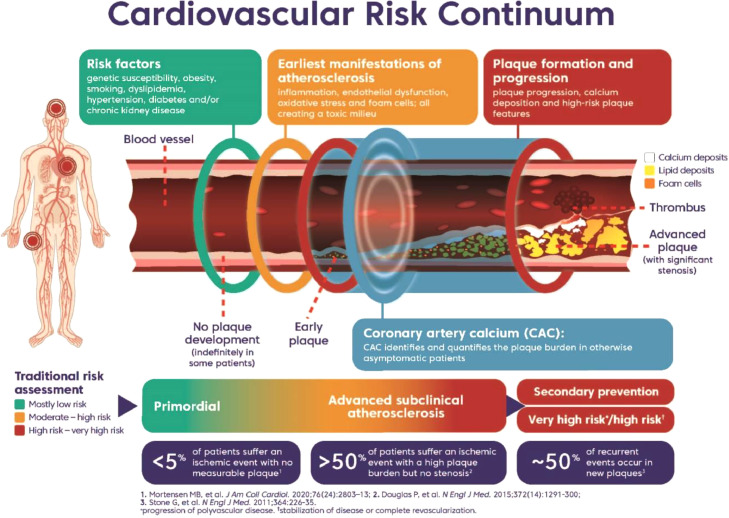
Conceptual graphic of the cardiovascular risk continuum, incorporating advanced subclinical atherosclerosis. The graphic may serve as a useful visual aid for physicians when discussing cardiovascular risk with patients and recognizes non-linear patterns of risk not reflected in traditional risk assessment algorithms [36], [37], [38].

Early in life (even in childhood), a combination of risk factors impacts the vascular endothelium to create a toxic milieu. This promotes the development and progression of atherosclerosis. During the primary prevention phase, there is an opportunity to halt or reverse some of these processes with changes in diet and lifestyle or with targeted control of risk factors such as hypertension, high LDL cholesterol, or hyperglycemia [39,40]. As the atherosclerosis progresses, which can take decades of an adult patient's life, the accumulation of lipid (the yellow areas in the graphic) and calcium (white areas) in plaque increases. In our model, plaque burden is the fundamental measure of disease risk [36]. Thereafter, an ischemic event may be caused by plaque rupture, erosion or progressive plaque stenosis. The graphic reflects and visualizes our increasing understanding that the majority of ischemic events do not take place in occluded vessels and that the global burden of atherosclerotic plaque is the best indicator of risk of an event rather than a single target lesion or stenosis.

The graphic is a tool that physicians can use when counseling patients. Integrating available measures of subclinical atherosclerosis in risk assessment, rather than categorizing patients strictly by the primary or secondary prevention categories, may enable patients to understand where they are along the cardiovascular risk continuum. Furthermore, it can be used as part of the clinician–patient risk discussion, during which the physician can illustrate the impact that their risk status may have on their future health and how lifestyle changes and preventive therapies might alter this trajectory. This strategy would better represent those patients who are at high risk of an event and highlight the need for more aggressive consideration of preventive measures. We strongly suggest renewed efforts for lifestyle modification and aggressive goal-based lowering of relevant risk factors in those people with advanced subclinical atherosclerosis who have not achieved optimal control of blood pressure, LDL cholesterol, or HbA1c, along with consideration for their treatment with low dose aspirin and/or emerging cardiometabolic therapies.

We believe that the simple visualization of the underlying and progressive processes involved in subclinical atherosclerosis presented here will encourage both clinicians and patients to think about risk in a different way. The graphic can be used to emphasize the importance of lifestyle and early risk modification, while uniquely drawing attention to advancing subclinical atherosclerosis, paving the way for a new paradigm of management and thereby reducing the risk of an ischemic event.

## Funding

No funding was provided for the development of this manuscript

## Disclosures statement

All authors are part of a Bayer AG global advisory board that convene to discuss risk assessment and preventive pharmacotherapy in primary and secondary prevention. MB and FS declare an investigator-initiated grant funding from Bayer AG. The remaining authors declare that they have no other conflicts of interest.

## CRediT authorship contribution statement

**Michael J. Blaha:** Conceptualization, Data curation, Formal analysis, Writing – original draft, Writing – review & editing. **Magdy Abdelhamid:** Conceptualization, Data curation, Formal analysis, Writing – original draft, Writing – review & editing. **Francesca Santilli:** Conceptualization, Data curation, Formal analysis, Writing – original draft, Writing – review & editing. **Zhongwei Shi:** Conceptualization, Data curation, Formal analysis, Writing – original draft, Writing – review & editing. **Dirk Sibbing:** Conceptualization, Data curation, Formal analysis, Writing – original draft, Writing – review & editing.

## Declaration of Competing Interest

The authors declare that they have no known competing financial interests or personal relationships that could have appeared to influence the work reported in this paper.

## References

[1] Roth G.A., Mensah G.A., Johnson C.O. (2020). GBD-NHLBI-JACC global burden of cardiovascular diseases writing group. Global burden of cardiovascular diseases and risk factors, 1990-2019: update from the GBD 2019 study. J Am Coll Cardiol.

[2] American College of Cardiology. ASCVD Risk Estimator Plus. Available at: http://tools.acc.org/ascvd-risk-estimator-plus/#!/calculate/estimate/ (accessed 24 June 2022).

[3] European Association of Preventive Cardiology. HeartScore. Available at: www.heartscore.org (accessed 24 June 2022).

[4] Javaid A., Dardari Z.A., Mitchell J.D. (2022). Distribution of coronary artery calcium by age, sex and race among patients Q1 30-45 years old. J Am Coll Cardiol.

[5] Santilli F., Zaccardi F., Liani R. (2020). *In vivo* thromboxane-dependent platelet activation is persistently enhanced in subjects with impaired glucose tolerance. Diabetes Metab Res Rev.

[6] Damman O.C., Bogaerts N.M.M., van den Haak M.J., Timmermans D.R.M. (2017). How lay people understand and make sense of personalized disease risk information. Health Expect.

[7] Sinning C., Makarova N., Völzke H. (2021). Association of glycated hemoglobin A1c levels with cardiovascular outcomes in the general population: results from the BiomarCaRE (Biomarker for Cardiovascular Risk Assessment in Europe) consortium. Cardiovasc Diabetol.

[8] Schlesinger S., Neuenschwander M., Barbaresko J. (2022). Prediabetes and risk of mortality, diabetes-related complications and comorbidities: umbrella review of meta-analyses of prospective studies. Diabetologia.

[9] Khaw K.T., Wareham N., Luben R. (2001). Glycated haemoglobin, diabetes, and mortality in men in Norfolk cohort of European prospective investigation of cancer and Nutrition (EPIC-Norfolk). BMJ.

[10] Esper R.J., Nordaby R.A., Vilariño J.O. (2006). Endothelial dysfunction: a comprehensive appraisal. Cardiovasc Diabetol.

[11] Toth P.P. (2008). Subclinical atherosclerosis: what it is, what it means and what we can do about it. Int J Clin Pract.

[12] Ahmadi A., Argulian E., Leipsic J. (2019). From subclinical atherosclerosis to plaque progression and acute coronary events: JACC state-of-the-art review. J Am Coll Cardiol.

[13] Dzaye O., Razavi A.C., Blaha M.J., Mortensen MB. (2021). Evaluation of coronary stenosis versus plaque burden for atherosclerotic cardiovascular disease risk assessment and management. Curr Opin Cardiol.

[14] Singh A., Collins B.L., Gupta A. (2018). Cardiovascular risk and statin eligibility of young adults after an MI: partners YOUNG-MI Registry. J Am Coll Cardiol.

[15] Nucifora G., Bax J.J., van Werkhoven J.M. (2011). Coronary artery calcium scoring in cardiovascular risk assessment. Cardiovasc Ther.

[16] Mohan J., Bhatti K., Tawney A. (2022). StatPearls.

[17] Greenland P., Blaha M.J., Budoff M.J. (2018). Coronary calcium score and cardiovascular risk. J Am Coll Cardiol.

[18] Greenland P., Lloyd-Jones D.M. (2022). Role of coronary artery calcium testing for risk assessment in primary prevention of atherosclerotic cardiovascular disease: a review. JAMA Cardiol.

[19] Dzaye O., Razavi A.C., Dardari Z.A. (2021). Modeling the recommended age for initiating coronary artery calcium testing among at-risk young adults. J Am Coll Cardiol.

[20] Bergström G., Persson M., Adiels M. (2021). Prevalence of subclinical coronary artery atherosclerosis in the general population. Circulation.

[21] Nasir K., Cainzos-Achirica M., Valero-Elizondo J. (2022). coronary atherosclerosis in an asymptomatic U.S. population: Miami heart study at Baptist health south Florida. JACC Cardiovasc Imaging.

[22] Agha A.M., Pacor J., Grandhi G.R. (2022). The prognostic value of CAC zero among individuals presenting with chest pain: a meta-analysis. JACC Cardiovasc Imaging.

[23] Celermajer DS. (2005). Primary and a half prevention: can we identify asymptomatic subjects with high vascular risk?. J Am Coll Cardiol.

[24] Gatto L., Prati F. (2020). Subclinical atherosclerosis: how and when to treat it?. Eur Heart J Suppl.

[25] Visseren F.L.J., Mach F., Smulders Y.M. (2021). 2021 ESC Guidelines on cardiovascular disease prevention in clinical practice: developed by the Task Force for cardiovascular disease prevention in clinical practice with representatives of the European Society of Cardiology and 12 medical societies With the special contribution of the European Association of Preventive Cardiology (EAPC). Eur Heart J.

[26] Feldman D.I., Michos E.D., Stone N.J. (2020). Same evidence, varying viewpoints: three questions illustrating important differences between United States and European cholesterol guideline recommendations. Am J Prev Cardiol.

[27] Davies M.J., D'Alessio D.A., Fradkin J. (2018). A consensus report by the American Diabetes Association (ADA) and the European Association for the Study of Diabetes (EASD). Diabetes Care.

[28] Kotseva K., De Backer G., De Bacquer D. (2019). Lifestyle and impact on cardiovascular risk factor control in coronary patients across 27 countries: results from the European Society of Cardiology ESC-EORP EUROASPIRE V registry. Eur J Prev Cardiol.

[29] Yano Y., O'Donnell C., Kuller L. (2017). Association of coronary artery calcium score vs age with cardiovascular risk in older adults: an analysis of pooled population-based studies. JAMA Cardiol.

[30] Houts P.S., Doak C.C., Doak L.G. (2006). The role of pictures in improving health communication: a review of research on attention, comprehension, recall, and adherence. Patient Educ Couns.

[31] Blaha M.J. (2016). Personalizing treatment: between primary and secondary prevention. Am J Cardiol.

[32] de Lemos J.A. (2022). Navar AM. A life-course approach to cardiovascular disease prevention. Nat Med.

[33] Mortensen M.B., Cainzos-Achirica M., Steffensen F.H. (2022). Association of coronary plaque with low-density lipoprotein cholesterol levels and rates of cardiovascular disease events among symptomatic adults. JAMA Netw Open.

[34] Raitakari O., Pahkala K., Magnussen C.G. (2022). Prevention of atherosclerosis from childhood. Nat Rev Cardiol.

[35] Jacobs D.R., Woo J.G., Sinaiko A.R. (2022). Childhood cardiovascular risk factors and adult cardiovascular events. N Engl J Med..

[36] Mortensen M.B., Dzaye O., Steffensen F.H. (2020). Impact of plaque burden versus stenosis on ischemic events in patients with coronary atherosclerosis. J Am Coll Cardiol.

[37] Douglas P.S., Hoffmann U., Patel M.R. (2015). Outcomes of anatomical versus functional testing for coronary artery disease. N Engl J Med.

[38] Stone G.W., Maehara A., Lansky A.J. (2011). A prospective natural-history study of coronary atherosclerosis. N Engl J Med.

[39] Volpe M., Gallo G., Modena M.G. (2022). Updated recommendations on cardiovascular prevention in 2022: an executive document of the Italian Society of Cardiovascular Prevention. High Blood Press Cardiovasc Prev..

[40] Villines T.C., Rodriguez Lozano P. (2020). Transitioning from stenosis to plaque burden in the cardiac CT era: the changing risk paradigm. J Am Coll Cardiol.

